# Rethinking Ischemic Acute Kidney Injury as Nephrotoxicity

**DOI:** 10.1093/function/zqae020

**Published:** 2024-04-17

**Authors:** David M Pollock

**Affiliations:** Section of CardioRenal Physiology & Medicine, Division of Nephrology, Department of Medicine, University of Alabama at Birmingham, Birmingham, AL 35233, USA

As scientists, we all believe that we are open to new ideas and novel mechanisms that contribute to pathogenesis of disease—but are we? When it comes to what we now refer to as acute kidney injury (AKI), we have gained tremendous insights into cellular processes that are impacted by a prolonged reduction in kidney perfusion associated with a decrease in glomerular filtration rate (GFR). Most of the work relative to ischemic AKI is focused on the premise that prolonged hypoxia cause injury to the tubular epithelium, putting the kidney at continued risk. However, the work from McLarnon and colleagues in the recent issue of *Function* provides an important example of why the complexities of AKI cannot be fully understood without considering the basic anatomy of physiology of the kidney, especially the role of renal hemodynamics.^1^

The problem of AKI has always been with us but did not gain wide attention until after World War II and the recognition of AKI associated with crush injuries.^2^ Work in this area also benefitted from significant progress led by several pioneering scientists and physicians. This includes the work of the Dutch scientist, Willem Kolff, who developed the first dialysis machine amid the chaos of the war and Joseph E. Murray, the military surgeon turned academic who took on the challenge of the first kidney transplant during the post-war expansion of biomedical research. Since this time, we have managed to prolong life of those who go on to develop chronic kidney disease albeit without a hope for a cure for those who are unable to receive a viable transplant.

Efforts to develop specific therapies to reduce or reverse AKI have met with little success. The initial approaches are varied often depending on where the nephrologist trained or their own anecdotal experience. Although many may manage relatively normal renal function after an episode of AKI, they remain at higher risk for additional kidney and cardiovascular complications. Much of the effort for initial treatment of AKI was designed to improve perfusion to alleviate what is believed to be an issue of hypoxic injury as well as increasing filtration to “flush” out tubular debris thought to arise primarily from injured tubules. Kidney-specific vasodilation with low-dose dopamine was one such approach that is at times still used. Fluid loading is thought to have potential benefit to maintain tubular flow and prevent oliguria, but this is often problematic for co-morbidities such as congestive heart failure.

The most common form of AKI arises from what is viewed as ischemic injury, which results from protracted reductions in renal perfusion that can occur during conditions such as cardiosurgical procedures or shock. Nephrotoxic kidney injury can also result from therapeutics such as contrast media, chemotherapeutic agents, and endogenous compounds such as hemoglobin or myoglobin that can be toxic to renal tubular epithelium. Model systems typically study these mechanisms in isolation, but McLarnon et al. provide compelling evidence for a key nephrotoxic component of ischemic injury that requires us to rethink the dogma of renal medullary hypoxic injury following renal ischemia.

Since the identification of a renal cortical-medullary oxygen gradient in the 1980s, tubular injury from ischemia, which is largely localized to the outer medulla of the kidney, has been thought to be primarily due to hypoxic cellular injury.^3^ Focus on hypoxia as the primary source of renal injury, however, does not explain much of the pathology of ischemic kidney injury^4^ and is not supported by evidence of selective cortical, rather than medullary ischemia, in AKI in humans.^5^

Red blood cell trapping, which presents as the expansion of the outer-medullary capillary plexus with tightly packed red blood cells, has long been recognized as a hallmark of ischemic kidney injury.^6,7^ While a number of studies have demonstrated the link between red blood cell trapping and kidney injury, red blood cell trapping has been thought to promote outer-medullary injury largely by extending ischemic time to the medulla and prolonging tissue hypoxia.^8,9^ McLarnon et al. first investigated the hypothesis that red blood cell trapping may lead to toxic rather than hypoxic tubular injury in a rat model of arterial clamping with reperfusion. Arterial occlusion followed by reperfusion for 2 h resulted in outer-medullary red blood cell trapping and tubular injury. Importantly, these investigators identified erythrocytic proteins within both the obstructing distal tubular casts and the outer-medullary tubular cells themselves, suggesting uptake of potential nephrotoxic red cell components, including hemoglobin. To further investigate this hypothesis and delineate the effects of ischemia from red blood cell trapping, McLarnon used a rat model of arterial versus venous occlusion to compare the effects of ischemia with red blood cell trapping to ischemia without red blood cell trapping on tubular injury. Venous clamping, which mimics the venous obstruction of the medullary circulation that occurs during reperfusion of the kidney following a period of ischemia and promotes the accumulation of red blood cells in the outer-medullary circulation (red blood cell trapping),^10^ resulted marked tubular injury in as little as 45 min. Importantly, this injury was not present in the blood-free kidney with venous clamping. In contrast, renal injury following arterial occlusion without reperfusion for the same period resulted in minimal histological injury to the kidney. As warm ischemia time was the same for both kidneys, this confirmed that red blood cell trapping was leading to toxic, rather than hypoxic, tubular injury. Consistent with a key role for hemodynamics as a critical factor in mediating AKI, Afolabi et al. recently demonstrated that rhabdomyolysis-induced AKI was mitigated by reductions in renal vascular resistance and increased GFR produced by inhibiting the endothelin-1 system.^11^

The lesson here is 2-fold. First, the source of a large amount of renal injury to the outer medulla following ischemia is related to the toxic effects of red cell contents that appear to be extravasated across the capillary walls of the congested outer-medulla circulation and are then identified within the damaged renal tubular epithelium. Second, well-established findings demonstrating the presence of hemoglobin within renal tubules following ischemia appear to have been largely forgotten in much of the recent research into the complex etiology of ischemic AKI.^12–14^ This new evidence provides a novel explanation for these findings, including the presence of hemoglobin in the distal tubules and urine of patients with ischemic AKI, even in those without elevated levels of circulating hemoglobin in the blood.^12^ In a 1975 article on ischemic kidney injury in the *Journal of the American Medical Association*, Berman notes that “few syndromes have had as many names and periodic rediscoveries as acute tubular necrosis,” the parade of names illustrating the changing concepts of pathogenesis of ischemic AKI.^15^ Based on the evidence presented by McLarnon that red blood cell trapping mediates toxic injury to the outer medulla following kidney ischemia, it may be worth resurrecting the name “hemoglobinuric nephrosis,” at least in concept, to explain the pathogenesis of outer-medullary tubular injury in ischemic AKI.
